# Supplementary material for: The effect of photobiomodulation on histamine and Mucuna pruriens-induced pruritus, hyperknesis and alloknesis in healthy volunteers: A double-blind, randomized, sham-controlled study
Source: PLoS One. 2024 Jul 18;19(7):e0307034. doi: 10.1371/journal.pone.0307034 (PMC11257285; doi:10.1371/journal.pone.0307034)
Supplement: S1 File — (PDF) [file pone.0307034.s002.pdf]

The effect of low-level light therapy on histamine- and Mucuna pruriens-induced itching in healthy volunteers: A prospective, double-blind, randomized sham-controlled pilot study

Version 3.0 13.8.2021

Kordula Lang-Illievich[1] , Heike Schulze-Bauer, Gudrun Rumpold-Seitlinger[1] , Helmar Bornemann-Cimenti[1]

1 ... Medical University of Graz, Clinical Department of Special Anesthesiology, Pain and Intensive Care Medicine

Sponsor

Medical University of Graz

"Histamine/LLLT"

Examiner

Dr. Kordula Lang-Illievich MSc

Department of Special Anesthesiology, Pain and Intensive Care Medicine
Auenbruggerplatz 2
8036 Graz

Table of contents

Page
List of abbreviations ... 3
Responsibilities and addresses ... 4
Synopsis ... 5
1. Scientific background ... 8
2. Designation and description of the test product ... 8
3. justification for the design of the clinical trial ... 10
4. risks and benefits of the investigational device and the clinical trial ... 11
5. objectives and hypotheses of the clinical trial ... 11
6. structure of the clinical trial ... 13
6.1 General information ... 13
6.2 Products and comparative products ... 16
6.3 Examinees ... 16
6.4 Treatments ... 17
6.5 Specifications for monitoring ... 18
7 Statistical considerations ... 18
8 Data management ... 19
9. amendments to the clinical trial protocol ... 20
10 Deviations from the clinical trial protocol ... 20
11 Proof of use of the product ... 20
12 Legal basis ... 20
13 Procedure for obtaining the declaration of consent ... 21
14 Adverse events, undesirable effects of the product and product defects ... 21
15 Premature termination or suspension of the examination ... 22
15.1 Discontinuation of the study by one subject (drop-out) ... 23
15.2 Termination of the entire study ... 23
16 Publication policy and final report ... 23
17 References ... 23
18. signatures ... 25

List of abbreviations

**FPFV**	**First Patient First Visit**
**LPLV**	**Last Patient Last Visit**
**BASG**	**Federal Office for Safety in Health Care**
**IIS**	**Investigator Initiated Studies**
**LLLT**	**Low Level Light Therapy**

Responsibilities and addresses

Sponsor:
Medical University of Graz
Auenbruggerplatz 5
8036 Graz
Austria

Clinical investigator (according to § 64 MPG):
Dr. Kordula Lang-Illievich MSc
Senior physician
Department of Special Anesthesiology, Pain and Intensive Care Medicine
Auenbruggerplatz 5
8036 Graz
0316385-80966
Kordula.lang-illievich@medunigraz.at

Test center:
Medical University of Graz
Department of Special Anesthesiology, Pain and Intensive Care Medicine
Auenbruggerplatz 5
8036 Graz
0316385-80966
emergency contact details: 06645381996
Kordula.lang-illievich@medunigraz.at

The trial is funded purely by the Principal Investigator - there is no public funding available, nor is the trial industry-supported.

Synopsis

**Sponsor**	**Medical University of Graz**
**Title**	**The effect of low level light therapy (LLLT) on histamine- and Mucuna pruriens-induced pruritus in healthy volunteers: A prospective, double-blind, randomized, sham-controlled trial**
**Abbreviation**	Histamine/LLLT
**Target population (or indication)**	Healthy test subjects undergoing an itch model
**Study design**	Prospective, double-blind, randomized, sham-controlled
**Aims of the clinical trial**	**Primary objective of the audit**<br>• Testing whether LLLT changes the intensity of itching after histamine application compared to sham application.<br>**Secondary objectives of the audit**<br>• Testing whether therapy with LLL changes the intensity of itching after Mucuna pruriens application compared to sham application.<br>• Testing whether therapy with LLL changes the size of the flare area compared to sham therapy after histamine application.<br>• Testing whether therapy with LLL changes the size of the flare area compared to sham therapy after Mucuna pruriens application.<br>• Testing whether therapy with LLL changes the skin temperature compared to sham therapy after histamine application.<br>• Testing whether therapy with LLL changes the skin temperature compared to sham therapy after Mucuna pruriens application.<br>• Testing whether treatment with LLL changes the size of the alloknesia area compared to sham treatment after histamine application.<br>• Testing whether therapy with LLL changes the size of the alloknesia area compared to sham treatment after Mucuna pruriens application.<br>• Testing whether treatment with LLL changes the size of the hyperknesia area compared to sham treatment after histamine application.<br>• Testing whether treatment with LLL changes the size of the hyperknesia area compared to sham treatment after Mucuna pruriens application.

**Objectives (endpoints) of the clinical trial**	**Primary target figure**<br><br>Itching intensity<br><br>**Secondary targets**<br><br>Flare area<br>Skin temperature<br>Allocation<br>Hyperknezia
**Number of patients**	17
**Schedule**	Audit-related<br><br>Recruitment time:2 weeks<br>Planned start (FPFV): July 2021 Planned end (LPLV): July 2021<br><br>Patient-related<br><br>Treatment duration: 3 hours
**Inclusion criteria**	• Written informed consent of the person taking part<br>• Healthy test subjects aged 18-60<br>• Negative pregnancy test for test subjects
**Exclusion criteria**	• Known allergy or hypersensitivity to histamine or the African itch bean<br>• anamnestic presence of skin diseases<br>• Tattoos in the test area<br>• Neoplasia in the test area<br>• Pacemaker<br>• Pregnancy<br>• Epilepsy<br>• Piercings<br>• Fever<br>• Local acute infection, skin inflammation/rashes
**Medical device**	Trade name:<br><br>"Repuls 7"<br><br>Manufacturer:<br><br>REPULS Lichtmedizintechnik GmbH
**Treatment plan**	Subject recruitment/education

	Carrying out a pregnancy test on test subjects<br>• Application of the 1st itch model<br>• Recording the measurement parameters<br>• LLLT or sham treatment<br>• Recording the measurement parameters<br>One hour waiting time<br>• Application of the 2nd itching model<br>• Recording the measurement parameters<br>• LLLT or sham treatment<br>• Recording the measurement parameters<br>Statistical evaluation

1. Scientific background

Low-level light therapy (LLLT) is a method of photobiomodulation that involves the clinical application of light with wavelengths that are usually in the range of 600 to 1100 nm and have a typical power density of 5 mW/cm² to 5 W/cm².
In addition to promoting wound healing, clinical studies have also demonstrated the positive effects of LLLT on pain management,[1-4] which are attributed to a reduction in neurogenic inflammation and the associated neuronal sensitization. This has been proven by testing the presence of hyperesthesia or allodynia.[2] In the course of neurogenic inflammation, however, histamine liberation also occurs, which can be associated with itching.[5] In analogy to chronic pain, chronic itching is also frequently associated with somatosensory abnormalities: itching-associated dysesthesias, such as mechanical allocnesia and hyperknezia, can be seen as an indication of neuronal sensitization in pruritus patients.[6]

Oliviera et al. were already able to demonstrate an antipruretic effect of LLLT in a case observation of five burn victims.[7] This effect was also investigated in lichen planus patients using laser irradiation, whose photobiomodulative mechanisms are similar to those of LLLT.[8]

Itch models can be divided into histamine-induced and non-histamine-induced models. To date, there has been no structured study on the antipruritic effect of LLLT, and the extent to which the mechanism that triggers itching has an influence on the suspected efficacy of LLLT is also unclear.

- *Summary of the risk-benefit assessment*

The intracutaneous application of histamine is a procedure routinely used in allergy diagnostics. There, histamine is used as a "positive control". Risks of histamine application alone cannot be found in the literature; however, data show that the risk of systemic side effects with intracutaneous allergy tests is 0.008%.[14] As a number of allergens are tested simultaneously with histamine administration, it can be assumed that the isolated risk of histamine is significantly lower.

The African itch bean is widely used for the production of itching powder. No systemic side effects have been reported for the Cowhagen model in the literature to date.

LLLT is described in the literature as having no side effects.[2, 15-17]

The application is carried out by specialists in anesthesiology and intensive care medicine in rooms with facilities for monitoring and emergency care.

2. Designation and description of the test product

a) Overview description of the test product and its intended use.

The REPULS 7 works with cold red light in the wavelength range of 630 nm. This form of radiation works at a wavelength that enables a significantly greater penetration depth than UV light. Due to the high power density and non-linearity of the dipole forces, the excitation of organic molecules, which serve as messenger substances, results in a doubling of the frequency and thus a halving of the wavelength in the tissue. This stimulates the resonance of the messenger substance and changes it through the resulting UV resonance oscillation at the respective location in the tissue in such a way that cleavage products are formed and transported away via the bloodstream. This allows inflammatory changes to subside.
The REPULS 7 is used to promote wound healing (irradiation of wounds only with non-contact application and use of the distance ring) and to promote scar healing.

b) Details of the manufacturer of the product.

REPULS Lichtmedizintechnik GmbH, Lemböckgasse 61 1230 Vienna

c) Name or number of the model/type number including the software version and, if available, the accessories to enable complete identification.

Repuls7
Serial number: 170670829

d) Description of how traceability can be achieved during and after testing, e.g. assignment of lot numbers, batch numbers or serial numbers.

The Clinic for Special Anesthesiology Pain and Intensive Care Medicine has access to 2 devices, which will also be used in the study. However, only one device (serial number: 170670829) will be put into operation. The other device (serial number: 131270190) is only used for sham irradiation.

e) Intended purpose of the investigational device in the planned clinical trial.

In the study planned here, the influence of LLLT on both histaminergic and non-histaminergic itching in healthy volunteers is to be tested.

f) Populations and indications for which the investigational product is intended.

The REPULS 7 is used to promote wound healing (irradiation of wounds only with non-contact application and use of the distance ring) and to promote scar healing.

g) Description of the investigational device, including all its materials that will come into contact with the tissues or body fluids (this must include details of medicinal products, human or animal tissues or their derivatives or other biologically active substances).

- Irradiation unit (article number 1003): This component is the heart of the device. The irradiation unit contains the irradiation diodes and all parts required for user interaction: digital display, buttons for inputs. 2.

- Handle (article number 2006): The handle is the key element for starting up the appliance. The appliance cannot be switched on without this part. The handle must only be accessible to authorized persons!
- Power supply unit (item number 2020): The power supply unit supplies the REPULS 7 with power.
- Safety goggles (article number 2001): The enclosed safety goggles must be used during irradiation.
- Spacer ring (article number 2018): The spacer ring serves as glare protection for non-contact applications (treatment of wounds).

h) *Overview of the training and experience required to use the test product.*

Training for the user is required before initial commissioning. According to the manufacturer, this includes the following content:

- Technical data
- Device operation & setting
- Application guidelines & treatment principles
- Safety & hazard information
- Error handling
- Maintenance & care

A training document covering this content, which is used for personal instruction, is provided to each user by the manufacturer or can be obtained from the manufacturer. Successful training on the device is confirmed by a training log. The current version of the training protocol_Repuls-7 is available and contains confirmation of the training of the above-mentioned contents.

i) *Description of the specific medical or surgical procedures associated with the use of the investigational product.*

There are no specific medical or surgical procedures associated with the use of the investigational product.

3. rationale for the design of the clinical trial

a) *assessment of the results of the relevant pre-clinical testing/evaluation conducted to justify the use of the investigational device in human subjects; and*

Clinical studies have demonstrated the positive effects of LLLT on both wound healing and pain management,[1-4] which are attributed to a reduction in neurogenic inflammation and the associated neuronal sensitization. LLLT can therefore also be used for acute and chronic pain.

b) *Assessment of clinical data relevant to the proposed clinical trial.*

In the course of neurogenic inflammation, histamine liberation also occurs, which can be associated with itching. The authors of this study therefore also expect a positive effect of LLLT on itching.

4. risks and benefits of the investigational device and the clinical trial

a) *Anticipated/foreseeable clinical benefit.*

The findings expected from the study presented are highly relevant for the assessment of the medical benefits of LLLT in itch therapy, as such quantitative statements have not been available in the literature to date. It is therefore to be expected that this study can make a significant contribution to a well-founded therapeutic recommendation.

b) *Anticipated/foreseeable adverse effect(s) of the product.*

LLLT is described as having no side effects.
The manufacturer has also not described any undesirable effects.

c) *Risks associated with participation in the clinical trial*

As only healthy volunteers are used for testing and LLLT is described as having no side effects, no risks are to be expected with regard to LLLT.
With regard to histamine and Mucuna pruriens application, temporary itching and slight reddening of the skin (intended for study purposes) occur. These remain localized to the area of histamine application and can last up to 2.5 hours.

d) *Possible interactions with concomitant medical treatments*

No interactions are to be expected.

e) *Measures to be taken to control or mitigate the risks*

None

f) *Risk/benefit justification.*

According to the current state of knowledge, a health risk for the participating test subjects can in fact be ruled out: No adverse effects are described in the literature. Thus, according to current knowledge, a risk from LLL irradiation is not to be expected for a short single application within the study. [18]

5. objectives and hypotheses of the clinical trial

a) *Primary and secondary goals.*

This study will provide the first quantitative analysis under controlled conditions of the potential influence of LLLT on both histaminergic and non-histaminergic pruritus, allocnesia and hyperknezia.

The following questions were to be answered in this study:

- Can therapy with LLL reduce the intensity of itching after histamine application ?
- Can therapy with LLL reduce the intensity of non-histaminergic itching after Mucuna pruriens application?
- Does therapy with LLL have an influence on neurogenic inflammation in the histaminergic itch model?
- Does treatment with LLL have an effect on neurogenic inflammation in the non-histaminergic itch model?
- Does treatment with LLL have an influence on the size of the alloknesia area in histaminergic pruritus?
- Does treatment with LLL have an influence on the size of the area of alloknesia in non-histaminergic pruritus?
- Does treatment with LLL have an influence on the size of the hyperknea area in histaminergic pruritus?
- Does treatment with LLL have an influence on the size of the hyperknea area in non-histaminergic pruritus?

b) Primary and secondary hypotheses to be accepted or rejected by statistical data from the clinical trial.

Primary hypothesis:
Therapy with LLL changes the intensity of itching after histamine application compared to sham application.

Secondary hypotheses:

- Therapy with LLL changes the intensity of itching after Mucuna pruriens application compared to sham application.
- Therapy with LLL changes the size of the flare area compared to sham therapy after histamine application.
- Therapy with LLL changes the size of the flare area compared to sham therapy after Mucuna pruriens application.
- Therapy with LLL changes the skin temperature compared to sham therapy after histamine application.
- Therapy with LLL changes the skin temperature compared to sham therapy after Mucuna pruriens application.
- Treatment with LLL changes the size of the alloknesia area compared to sham treatment after histamine application.

- Treatment with LLL changes the size of the alloknesia area compared to sham treatment after application of Mucuna pruriens.
- Treatment with LLL changes the size of the hyperknesia area compared to sham treatment after histamine application.
- Treatment with LLL changes the size of the hyperknesia area compared to sham treatment after Mucuna pruriens application.

6. structure of the clinical trial

6.1 General information

a) *Description of the type of trial to be conducted (e.g. double-blind comparative trial, parallel design, with or without comparator product group) with justification for the selection;*

The trial will be prospective, double-blind, randomized and sham-controlled.

b) *Description of measures to be taken to minimize or avoid bias; including randomization and blinding.*

The side of the LLLT or sham treatment is determined by a computer-generated random list "www.randomizer.at". The treatment is carried out by a person who is not involved in the further conduct of the study.
The test subjects are blinded to the irradiation side by audiovisual shielding (swimming goggles with blackened lenses, hearing protection). The subjects do not have access to the online randomization and are not in the same room during the irradiation. As a result, they are also blinded to the radiation side.

c) *Primary and secondary endpoints with the rationale for their selection and measurement.*

1. Histaminergic itching is determined using a visual analog scale (0-100)
2. Non-histaminergic itching is determined using a visual analog scale (0-100)
3. The neurogenic inflammation of the histaminergic itchrecording the flare area and measuring the skin temperature.
4. The neurogenic inflammation of non-histaminergic itching is determined by recording the flare area and measuring the skin temperature
5. An area of alloknesia is tested in the histaminergic itch model using cotton swabs
6. An area of allocnesia is tested in the non-histaminergic itch model using cotton swabs
7. A hyperknea area istested in the histaminergic itch modelwith a von Frey filament.
8. A hyperknea area is tested in the non-histaminergic itch model with a Frey filament

Itch models can be divided into histamine-induced and non-histamine-induced models. To date, there has been no structured study on the antipruritic effect of LLL, and the extent to which the mechanism that triggers itching has an influence on the suspected efficacy of LLL is also unclear.

In analogy to chronic pain, chronic itching is also frequently associated with somatosensory abnormalities. Itch-associated dysesthesias, such as mechanical allocnesia and hyperknezia, can be seen as an indication of neuronal sensitization in pruritus patients[6] and thus provide indications as to whether the antipruretic effect can also be expected in chronic pruritus.

d) *Procedure and choice of time for evaluating, recording and analyzing the variables.*

Parameters collected

Intensity of the itching
The intensity of the itching was measured using a visual analog scale (0-100), which the test subjects had previously been trained to use.

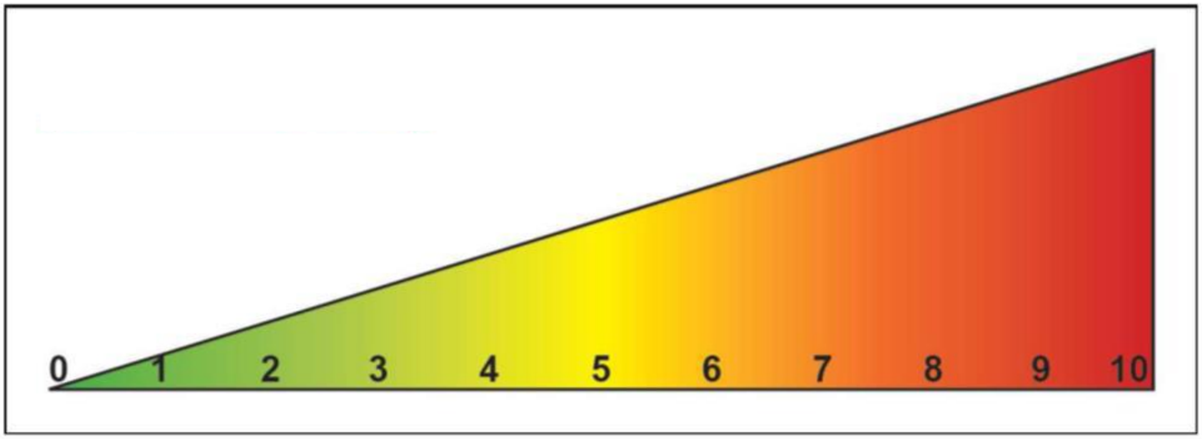

Figure 4: VAS scale of the LKH University Hospital Graz

Flare area
The size of the flare area is calculated using ImageJ software (National Institutes of Health, Bethesda, USA; ImageJ is in the public domain: http://rsb.info.nih.gov/ij).

Skin temperature
The skin temperature is measured in the center of the flare area using a disposable Corpuls C3 temperature probe.

Allocation
The next step is to measure the alloknesis distance using a cotton swab, which is used to stroke the skin along 8 radial paths in a series of short strokes. Stroking begins 10 cm from the puncture site / center of the cowhage application and continues in the direction of the puncture site until

the subject reports a feeling of itching during the stroking or until the strokes reach within 1 cm of the puncture site.[12] The 8 measurements are averaged.

Hyperknezia
The hypeknesia is then recorded using a von Frey filament: this is also placed 10 cm from the puncture site/center of the cowhage application centripetally along 8 radial lines at 0.5 cm intervals. Subjects are instructed to ignore the initial stinging and only assess the extent of itching on a visual analog scale. The 8 measurements are averaged.[12]

Time sequence
After subject recruitment, information about the planned trial is provided by a medical study staff member, followed by the signing of the Informed Consent Form by the subject and the study staff member. Subjects undergo a pregnancy test.
This is followed by computer-aided randomization.
Compliance with the time sequence is coordinated by a stopwatch with pre-programmed signals.
LLL is applied for 12 minutes using Repuls 7 (Repuls Lichtmedizintechnik GmbH, Vienna).

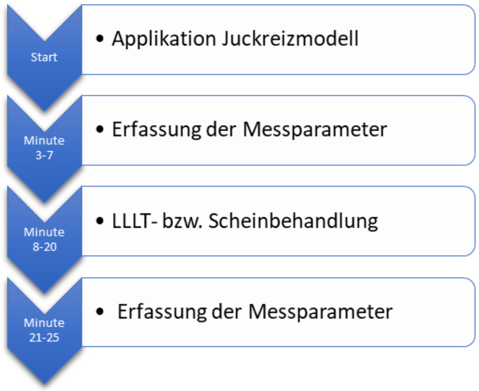

After one hour, repeat the algorithm with the other itch model

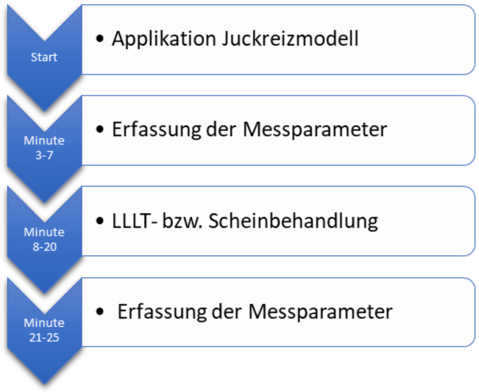

e) Any instruction for the exchange of test subjects

There will be no exchange of exam participants.

6.2 Products and comparative products

a) *Description of the exposure to the test or comparator products, if the latter are intended.*

The test subjects are exposed to the application of LLL for 2x12 minutes (histamine-induced and non-histamine-induced pruritus area). The device is placed 7 cm above the pruritus area using a spacer ring.
In sham treatment, the second device is also placed over the pruritus area using a spacer ring, but is not activated.

b) Justification for the choice of comparative products.
none

c) List of all other products or drug treatments to be used during the test.

The test subjects go through two itch models. For this purpose, the back is divided into 4 quadrants. The histamine model is applied to the two upper quadrants, the Mucuna pruriens model is applied to the two lower quadrants after a one-hour interval.

Histamine model
The methodology corresponds to the model described by Darsow et al.[10] After applying a drop of histamine gel (1% histamine dihydrochloride in 2.5% methylcellulose), the skin is punctured superficially using conventional blood lancets, as are commonly used in allergy diagnostics.[10] The application is always carried out by the same study employee in order to minimize variability in the application technique.

Cowhagen/Mucuna pruriens model
The plant hairs of Mucuna pruriens (African itch bean) are an established model for inducing histamine-independent itching. The methodology corresponds to the model described by Papoiu et al. An area of 2x2cm is covered with Hansaplast strips to prevent the plant hairs from spreading to the surrounding skin. In this skin area, 40 to 45 threads, which are previously counted under a microscope, are applied with micro-tweezers and gently rubbed in for 45 seconds.[11]

6.3 Examinees

a) *Inclusion criteria for the selection of* test *participants*

Healthy volunteers aged 18-60 years who are willing to participate after detailed information (signed informed consent). Female volunteers must have a negative pregnancy test.
.

b) Exclusion criteria for the selection of candidates.

- Known allergy or hypersensitivity to histamine or the African itch bean
- anamnestic presence of skin diseases
- Piercings
- Fever
- Tattoos in the test area
- Neoplasia in the test area
- Pregnancy
- Pacemaker
- Local acute infection, skin inflammation, rashes
- Epilepsy

c) Time of recording.

Signing of the informed consent

d) Expected total duration of the clinical trial.

3 weeks

e) Expected duration of participation of each participant.

3 hours

f) Required number of subjects to be included in the clinical trial.

17 test persons

g) Estimated time required to recruit this number of people (i.e. duration of recording).

The recruitment phase will last approximately 2 weeks. Recruitment will take place on the notice boards of the ÖH at the KF University Graz (main building, Resowi, Wall building).

6.4 Treatments

a) Description of all treatments administered to subjects during the clinical trial. subjects during the trial.

LLL is applied for 12 minutes using Repuls 7 (Repuls Lichtmedizintechnik GmbH, Vienna). The device is positioned 7 cm away from the skin using a distance ring.
The sham treatment is carried out with the same device and the same spacer ring without activating it.

b) Any known or foreseeable factors that may affect the results of the clinical trial or its interpretation. or their interpretation.

The results obtained in this study in a group of healthy volunteers and an experimental itch model with single application of LLLT can only be transferred to clinical application to a limited extent.

6.5 Specifications for monitoring

The auditor agrees that the person responsible for monitoring will carry out a data check in accordance with the monitoring plan to ensure that the data is collected satisfactorily and that the audit plan is adhered to.
Furthermore, he/she agrees to cooperate with this person and to provide him/her with all necessary information whenever required. This includes access to all documents related to the examination, including the original patient files relevant to the examination. One of the tasks of the investigator is to keep the patient file as complete as possible, i.e. to record information on medical history, concomitant illnesses, admission to the trial, dates of visits, results of examinations, medication dispensed and adverse events. The monitor is also enabled to perform the data review and comparison with the relevant patient records in accordance with the SOPs and ICH-GCP guidelines at the predetermined intervals to ensure compliance with the protocol and continuous recording of data. All original medical findings that are necessary as a source for the information in the CRF or in the database are checked. The trial participants have agreed to such a review by signing the declaration of consent.
The person responsible for monitoring is obliged to treat all information confidentially and to safeguard the fundamental right of the participating persons to integrity and protection of their privacy.

7 Statistical considerations

a) statistical planning, the procedure and the analytical methods,

The number of cases was calculated based on the following assumptions: In the publication by Arendt-Nielsen et al. itch severity was determined to be 32.9 ± 5.6. A reduction of more than 15% was defined as clinically relevant.

Statistical evaluation
Depending on the distribution, the statistical analysis is carried out using the 2-sided T-test for dependent samples or Wilcoxon test. The significance level alpha is defined as 0.05.

b) the sample size,

This results in a minimum sample size of 15 test subjects for a 2-sided T-test with linked samples. The sample size was calculated using G*Power 3.1.9.5.[13]

c) *the significance level and the informative value of the clinical trial,*

The significance level alpha was defined as 0.05, beta as 0.1.

d) *the expected failure rates,*

In order to be able to take drop-outs into account, the sample size was increased to 17 test subjects.

e) *the pass/fail criteria to be applied to the results of the examination,*
none

f) *if necessary, the measures for an interim evaluation,*
none

g) *the criteria for terminating the test for statistical reasons,*
none

h) *Instructions for the report on all deviations from the original statistical statistical planning,*
none

i) *the specification of the subgroups of the analysis,*
none

j) *Processes that take all data into account,*
none

k) *the handling of missing, unused, incorrect or misleading data, including defaults and withdrawals of individual subjects,*
none

l) *a justification for not considering individual pieces of information in the hypothesis testing, if applicable, and*
none

m) *for multi-center trials, the minimum and maximum number of subjects to be included for each trial site.*
none

8. data management

Each subject is assigned a study code. The study documents are kept locked away. Only authorized personnel have access to this data.

The data analysis is based only on the study codes.

The provisions of the General Data Protection Regulation are observed.

In accordance with the KaKuG, medical records are kept for 30 years.

9. changes to the clinical trial protocol

The vote of the ethics committee only covers the information contained in the application, i.e. it does not include extensions and amendments to the research project made at a later date. In the case of amendments, an amendment to the protocol is required, which must be signed by the investigator. Every change to the protocol must be added as an amendment to every protocol in circulation. The Ethics Committee must be notified of all substantial protocol amendments. For protocol amendments that are not exclusively of a formal nature and contain changes relevant to the *trial subject*, a new vote of the Ethics Committee must be obtained. Patients/subjects must be informed of any changes to the study conditions as part of the informed consent process.
Significant changes to the test plan must also be reported to the authority (BASG). The corresponding notification forms on the BASG website must be used for these notifications.

10. deviations from the clinical trial protocol

a) *Declaration stating that the auditor is not permitted to deviate from the CIP*.

Deviations from the trial protocol are not permitted, except in the case of urgent safety measures for the trial subjects. Such Urgent Safety Measures must be reported to the Ethics Committee/the BASG immediately after their implementation.

b) *Instructions for recording, reporting and analyzing deviations from the clinical clinical trial protocol.*

Test plan deviations are documented by the PI and a root cause analysis is carried out, on the basis of which measures are implemented to avoid the deviation in the future (corrective and preventive measures).

Deviations from the protocol that could have an impact on patient safety are reported to the ethics committee.

c) *Reporting requirements and time frame.*

If applicable, the report should be made promptly after the deviation becomes known.

11.

12 Legal basis

12.1
In addition to the Declaration of Helsinki (as amended), the following guidelines and laws must be observed when conducting the study: e.g.

- MPG as amended from time to time
- EN 14155
- ICH-GCP Guideline
- EU REGULATION (EU) 2017/745

12.2
In accordance with the Medical Devices Act, personal injury/legal protection insurance is taken out.

12.3 Vote of the Ethics Committee

The clinical trial may only be started if the approving opinion of the responsible ethics committee and the non-prohibition/approval of the responsible authority (BASG) have been received.

13. procedure for obtaining the declaration of consent

a) *Description of the general procedure for obtaining informed consent, including the procedure for providing subjects with new information when necessary.*

Subjects are informed by authorized study staff. The subjects have sufficient time to make their decision and ask questions. The subjects receive a copy of the informed consent form. If relevant new information becomes available, the subjects who are still in the study will be informed again.

14. adverse events, undesirable effects of the product and product defects

a) Definitions of adverse events

An **Adverse Event (AE)** means an adverse medical event, unanticipated illness or injury, or adverse clinical symptoms, including abnormal laboratory findings, in subjects, users, or others.

An **AE** can therefore be any adverse and unintended reaction (including an abnormal laboratory finding), symptom or transient condition, whether or not it is related to the investigational product.

An **adverse effect of the product** is an AE for which a causal relationship to the study product/test method is assumed.

b) Definitions of serious adverse events

A **serious adverse event (SAE**) is an adverse event that has one of the following consequences:

a) Death

b) serious deterioration in the subject's medical condition that resulted in any of the following: i) life-threatening illness or injury, ii) permanent physical injury or permanent impairment of a bodily function, iii) hospitalization or prolongation of hospitalization of the patient, iv) medical or

surgical intervention to prevent a life-threatening illness or injury or permanent physical injury or permanent impairment of a bodily function, v) chronic illness,

c) Fetal endangerment, fetal death or congenital physical or mental impairment or birth defects

A **serious adverse device event (SADE)** is an SAE that is considered to be causally related to the study product/test method.

c) Definition of product defects

'product defect' means an inadequacy in the identification, quality, durability, reliability, safety or performance of a test product, including malfunction, misuse or inadequacy of information provided by the manufacturer

e.) Documentation and reporting obligations in accordance with the Medical Device Regulation, Article 80

The sponsor keeps **complete records** of all of the following elements:

a) adverse events of all types identified in the clinical trial protocol as critical to the evaluation of the results of this clinical trial;
The occurrence of pain in the test area due to the application of LLLT would be decisive for the evaluation of the results.
b) all serious adverse events;
c) any product defect that could have led to serious adverse events in the absence of appropriate action or intervention or under less favorable circumstances;
(d) any new findings in relation to an event referred to in points (a) to (c).

The sponsor shall immediately **notify** all Member States in which the clinical trial is being conducted,

(a) any serious adverse event that has a causal relationship with the investigational device, comparator or test method or where a causal relationship appears reasonably possible,
b) any product defect that could have led to serious adverse events in the absence of appropriate action or intervention or under less favorable circumstances,
(c) any new findings in relation to an event referred to in points (a) and (b).

The deadline within which the notification must be made depends on the severity of the event. If this is necessary to ensure prompt reporting, the sponsor can first submit an incomplete report and then follow this up with the full report. The report is also sent to the evaluating ethics committee.

15. premature termination or suspension of the examination

Requirements for the follow-up of examination participants.

A follow-up of the test subjects is not planned.

15.1 Discontinuation of the study by one subject (drop-out)

One or more of the following circumstances may, for example, lead to an individual subject dropping out of the study (this subject is counted as a drop-out):
- Withdrawal of the subject's consent
- Non-tolerable adverse effects
- Violation of the study protocol
- Occurrence of an exclusion criterion
- Occurrence of a disease
- Pregnancy
- other circumstances that would jeopardize the subject's health if he or she continued to participate in the study.

15.2 Termination of the entire study

The investigator may discontinue the study at any time for the benefit and in the interest of the subjects if severe side effects or other unforeseeable circumstances occur.

16 Publication policy and final report

The study will be registered on Clinicaltrials.gov before it starts

All randomized and controlled clinical trials that recruit patients after July 1, 2005 must be registered in a publicly accessible database (e.g. www.clinicaltrials.gov) in order to be able to publish the trial results in renowned journals once they are available.

The final report must be submitted to the BASG/the Ethics Committee one year after completion of the study.

17. literature references

References

1. Esper, M.A., R.A. Nicolau, and E.A. Arisawa, The effect of two phototherapy protocols on pain control in orthodontic procedure--a preliminary clinical study Lasers Med Sci, 2011. 26(5): p. 657-63.
2. Lang-Illievich, K., et al, The Effect of Low-Level Light Therapy on Capsaicin-Induced Peripheral and Central Sensitization in Healthy Volunteers: A Double-Blinded, Randomized, Sham-ControlledTrial. Pain Ther, 2020.

3. Langella, L.G., et al, Photobiomodulation therapy (PBMT) on acute pain and inflammation in patients who underwent total hip arthroplasty-a randomized, triple-blind, placebo-controlled clinical trial Lasers in medical science, 2018. 33(9): p. 1933-1940.
4. , A.C.G., et al, Low-level laser and light-emitting diode therapy for pain control in hyperglycemic and normoglycemic patients who underwent coronary bypass surgery with internal mammary artery grafts: a randomized, double-blindstudy with follow-up. Photomedicine and laser surgery, 2016. 34(6): p. 244-251.
5. Rosa, A.C. and R. Fantozzi, The role of histamine in neurogenic inflammation Br J Pharmacol, 2013. 170(1): p. 38-45.
6. Andersen, H.H., et al, Alloknesis and hyperknesis-mechanisms, assessment methodology, and clinical implications of itch sensitization Pain, 2018. 159(7): p. 1185-1197.
7. de Oliveira, R.A., et al, Low-intensity LED therapy (658 nm) on burn healing: a series of cases Lasers Med Sci, 2018. 33(4): p729-735.
8. Ogrinc, U., et al, Efficacy of Non-ablative Laser Therapy for Lichen Sclerosus: A Randomized Controlled Trial J Obstet Gynaecol Can, 2019. 41(12): p. 1717-1725.
9. Andersen, H.H., et al, Nonhistaminergic and mechanical itch sensitization in atopic dermatitis Pain, 2017. 158(9): p. 1780-1791.
10. , U., et al, Correlations between histamine-induced wheal, flare and itch. Arch Dermatol Res, 1996. 288(8): p. 436-41.
11. Papoiu, A.D., et al, Cowhage-induced itch as an experimental model for pruritus. A comparative study with histamine-induced itch. PLoS One, 2011. 6(3): p. e17786.
12. , P., et al, Similar itch and nociceptive sensations evoked by punctate cutaneous application of capsaicin, histamine and cowhage Pain, 2009. 144(1-2): p. 66-75.
13. Faul, F., et al, Statistical power analyses using G* Power 3.1: Tests for correlation and regression analyses Behavior research methods, 2009. 41(4): p. 1149-1160.
14. Codreanu, F., et al, The risk of systemic reactions to skinprick-tests using food allergens: CICBAA data and literature review. Eur Ann Allergy Clin Immunol, 2006. 38(2): p. 52-4.
15. , H., The Repuls© depth radiator as an additional therapy option for patients with shoulder complaints.2012.
16. de Freitas, L.F. and M.R. Hamblin, Proposed mechanisms of photobiomodulation or low-level light therapy IEEE Journal of selected topics in quantum electronics, 2016. 22(3): p. 348-364.
17. Jajarm, H., et al, The effects of photodynamic and low-level lasertherapy for treatment of oral lichen planus-A systematic review and meta-analysis. Photodiagnosis Photodyn Ther, 2018. 23: p. 254-260.
18. Rayegani, S.M., et al, Safety and Effectiveness of Low-Level Laser Therapy in Patients With Knee Osteoarthritis: A Systematic Review and Meta-analysisJ Lasers Med Sci, 2017. 8(Suppl 1): p. S12-s19.

18. signatures

18.1

The signature confirms that the study will be conducted in accordance with ICH-GCP, the Declaration of Helsinki, national legislation and this study protocol.

Sponsor or its representative

Dr. Kordula Lang-Illievich **Graz, 3.6.2**

18.2

Examiner

I hereby confirm that I have read and understood this protocol and accept it in its entirety. I undertake to ensure that the persons brought into the study by my center are treated, observed and documented in accordance with the provisions of this study plan.

Dr. Kordula La Graz, 3.6.21

.
