# Supplementary material for: The effect of photobiomodulation on histamine and Mucuna pruriens-induced pruritus, hyperknesis and alloknesis in healthy volunteers: A double-blind, randomized, sham-controlled study
Source: PLoS One. 2024 Jul 18;19(7):e0307034. doi: 10.1371/journal.pone.0307034 (PMC11257285; doi:10.1371/journal.pone.0307034)
Supplement: S2 File — (PDF) [file pone.0307034.s003.pdf]

Der Effekt der Low Level Licht Therapie auf Histamin- und Mucuna pruriens induzierten Juckreiz bei gesunden Probanden: Eine prospektiv, doppelblind, randomisierte Schein-kontrollierte Pilotstudie

Version 3.0 13.8.2021

Kordula Lang-Illievich[1], Heike Schulze-Bauer, Gudrun Rumpold-Seitlinger[1], Helmar Bornemann-Cimenti[1]

1 … Medizinische Universität Graz, Klinische Abteilung für Spezielle Anästhesiologie, Schmerz und Intensivmedizin

Sponsor

Medizinische Universität Graz

„Histamin/LLLT“

Prüfer

Dr. Kordula Lang-Illievich MSc

Abteilung für Spezielle Anästhesiologie, Schmerz - und Intensivmedizin
Auenbruggerplatz 2
8036 Graz

Inhaltsverzeichnis

Seite
Verzeichnis der Abkürzungen 3
Verantwortlichkeiten und Anschriften 4
Synopsis 5
1. Wissenschaftlicher Hintergrund 8
2. Bezeichnung und Beschreibung des Prüfproduktes 9
3. Begründung für den Aufbau der klinischen Prüfung 11
4. Risiken und Nutzen des Prüfprodukts und der klinischen Prüfung 11
5. Ziele und Hypothesen der klinischen Prüfung 12
6. Aufbau der klinischen Prüfung 13
6.1 Allgemeines 13
6.2 Produkte und Vergleichsprodukte 16
6.3 Prüfungsteilnehmer 17
6.4 Behandlungen 18
6.5 Festlegungen für das Monitoring 18
7. Statistische Überlegungen 19
8. Datenmanagement 20
9. Änderungen am klinischen Prüfplan 20
10. Abweichungen vom klinischen Prüfplan 21
11. Verwendungsnachweis des Produkts 21
12. Rechtliche Grundlagen 21
13. Verfahren zum Einholen der Einverständniserklärung 22
14. Unerwünschte Ereignisse, unerwünschte Wirkungen des Produkts und Produktmängel 22
15. Vorzeitige Beendigung oder Aussetzen der Prüfung 24
15.1 Abbruch der Studie bei einem Probanden (Drop-out) 24
15.2 Abbruch der gesamten Studie 24
16. Veröffentlichungspolitik und Abschlussbericht 24
17. Literaturhinweise 24
18. Unterschriften 26

Verzeichnis der Abkürzungen

**FPFV**	**First Patient First Visit**
**LPLV**	**Last Patient Last Visit**
**BASG**	**Bundesamt für Sicherheit im Gesundheitswesen**
**IIS**	**Investigator Initiated Studies**
**LLLT**	**Low Level Light Therapie**

Verantwortlichkeiten und Anschriften

Sponsor:
Medizinische Universität Graz
Auenbruggerplatz 5
8036 Graz
Österreich

Klinischer Prüfer (gemäß § 64 MPG):
Dr. Kordula Lang-Illievich MSc
Oberärztin
Abteilung für Spezielle Anästhesiologie, Schmerz – und Intensivmedizin
Auenbruggerplatz 5
8036 Graz
0316385-80966
Kordula.lang-illievich@medunigraz.at

Prüfzentrum:
Medizinische Universität Graz
Abteilung für Spezielle Anästhesiologie, Schmerz – und Intensivmedizin
Auenbruggerplatz 5
8036 Graz
0316385-80966
emergency contact details: 06645381996
Kordula.lang-illievich@medunigraz.at

Die Finanzierung der Prüfung erfolgt rein über den Principal Investigator – es stehen weder öffentliche Fördergelder zu Verfügung, noch ist die Prüfung industriegestützt.

Synopsis

**Sponsor**	**Medizinische Universität Graz**
**Titel**	**Der Effekt der Low Level Licht Therapie (LLLT) auf Histamin- und Mucuna pruriens induzierten Juckreiz bei gesunden Probanden: Eine prospektiv, doppelblind, randomisierte, Schein-kontrollierte Prüfung**
**Kurzbezeichnung**	Histamin/LLLT
**Zielpopulation (oder Indikation)**	Gesunde Proband*innen, die sich einem Juckreizmodell unterziehen
**Studiendesign**	Prospektiv, doppelblind, randomisiert, Schein-kontrolliert
**Ziele der klinischen Prüfung**	**Primäres Ziel der Prüfung**<br>• Prüfung, ob LLLT im Vergleich zu Scheinanwendung die Intensität des Juckreizes nach Histaminapplikation verändert.<br>**Sekundäre Ziele der Prüfung**<br>• Prüfung, ob die Therapie mit LLL im Vergleich zu Scheinanwendung die Intensität des Juckreizes nach Mucuna pruriens Applikation verändert.<br>• Prüfung, ob die Therapie mit LLL im Vergleich zu Scheintherapie nach Histamin-Applikation die Größe des Flareareals verändert.<br>• Prüfung, ob die Therapie mit LLL im Vergleich zu Scheintherapie nach Mucuna pruriens Applikation die Größe des Flareareals verändert.<br>• Prüfung, ob die Therapie mit LLL im Vergleich zu Scheintherapie nach Histamin-Applikation die Hauttemperatur verändert.<br>• Prüfung, ob die Therapie mit LLL im Vergleich zu Scheintherapie nach Mucuna pruriens Applikation die Hauttemperatur verändert.<br>• Prüfung, ob die Therapie mit LLL im Vergleich zu Scheinbehandlung nach Histamin-Applikation die Größe des Alloknesieareals verändert.<br>• Prüfung, ob die Therapie mit LLL im Vergleich zu Scheinbehandlung nach Mucuna pruriens Applikation die Größe des Alloknesieareals verändert.<br>• Prüfung, ob die Therapie mit LLL im Vergleich zu Scheinbehandlung nach Histamin-Applikation die Größe des Hyperknesieareals verändert.<br>• Prüfung, ob die Therapie mit LLL im Vergleich zu Scheinbehandlung nach Mucuna pruriens Applikation die Größe des Hyperknesieareals verändert.

**Zielgrößen (Endpunkte) der klinischen Prüfung**	**Primäre Zielgröße**<br><br>Juckreizintensität<br><br>**Sekundäre Zielgrößen**<br><br>Flare Areal<br>Hauttemperatur<br>Alloknesie<br>Hyperknesie
**Patientenzahl**	17
**Zeitplan**	Prüfungsbezogen<br><br>Rekrutierungszeit:2 Wochen<br>Geplanter Beginn (FPFV): Juli 2021<br>Geplantes Ende (LPLV): Juli 2021<br><br>Patientenbezogen<br><br>Behandlungsdauer: 3 Stunden
**Einschluss-kriterien**	• Schriftliche Einwilligung der teilnehmenden Person nach erfolgter Aufklärung<br>• Gesunde Proband*innen im Alter von 18-60<br>• Bei Probandinnen negativer Schwangerschaftstest
**Ausschluss-kriterien**	• Bekannte Allergie oder Überempfindlichkeit gegen Histamin oder die afrikanische Juckbohne<br>• anamnestische Vorliegen von Hautkrankheiten<br>• Tattoos im Prüfgebiet<br>• Neoplasie im Prüfgebiet<br>• Schrittmacher<br>• Schwangerschaft<br>• Epilepsie<br>• Piercings<br>• Fieber<br>• Lokal akuter Infekt, Hautentzündungen/-ausschläge
**Medizinprodukt**	Handelsname:<br><br>„Repuls 7“<br><br>Hersteller:

	REPULS Lichtmedizintechnik GmbH
**Behandlungsplan**	Probandenrekrutierung/Aufklärung<br><br>Durchführung eines Schwangerschaftstests bei Probandinnen<br><br>• Applikation des 1.Juckreizmodells<br>• Erfassung der Messparameter<br>• LLLT- bzw. Scheinbehandlung<br>• Erfassung der Messparameter<br><br>Eine Stunde Wartezeit<br><br>• Applikation des 2.Juckreizmodells<br>• Erfassung der Messparameter<br>• LLLT- bzw. Scheinbehandlung<br>• Erfassung der Messparameter<br><br>Statistische Auswertung

1. Wissenschaftlicher Hintergrund

Low-Level-Light Therapie (LLLT) ist eine Methode der Photobiomodulation, bei der es sich um die klinische Anwendung von Licht mit Wellenlängen handelt, die üblicherweise im Bereich von 600 bis 1100 nm liegen und eine typische Leistungsdichte von 5 mW/cm² bis 5 W/cm² besitzen.
In klinischen Studien konnten neben der Förderung der Wundheilung auch positive Effekte von LLLT auf die Schmerzverarbeitung nachgewiesen werden,[1-4] welche auf eine Reduktion der neurogenen Inflammation und der damit assoziierten neuronalen Sensibilisierung zurückgeführt werden. Bewiesen wurde dies über die Prüfung einer vorliegenden Hyperästhesie bzw. Allodynie.[2] Im Zuge einer neurogenen Inflammation kommt es aber auch zu einer Histaminliberation, welche mit Juckreiz vergesellschaftet sein kann.[5] Chronischer Juckreiz ist in Analogie zu chronischem Schmerz ebenso häufig mit somatosensorischen Auffälligkeiten vergesellschaftet: Juckreiz-assoziierte Dysästhesien, wie die mechanische Alloknesie und Hyperknesie können als Hinweis für eine neuronale Sensibilisierung bei Prurituspatient*innen gesehen werden.[6]

Oliviera et al. konnten einen antipruretischen Effekt durch LLLT bereits an einer Fallbeobachtung an fünf Verbrennungsopfern zeigen.[7] Durch die Bestrahlung mit Laser, dessen photobiomodulative Mechanismen den Mechanismen der LLLT ähneln, wurde dieser Effekt ebenso an Lichen planus Patient*innen untersucht.[8]

Juckreizmodelle lassen sich in histamininduzierte und nicht-histamininduzierte unterscheiden. Bisher gab es noch keine strukturierte Untersuchung über die antipruretische Wirkung von LLLT, ebenso ist offen, inwieweit der Juckreiz auslösende Mechanismus einen Einfluss auf die suszipierte Wirksamkeit von LLLT hat.

• *Zusammenfassung der Risiko-Nutzen-Abschätzung*

Die intrakutane Histamin-Applikation ist ein in der Allergiediagnostik routinemäßiges eingesetztes Verfahren. Dort wird Histamin als „Positivkontrolle“ verwendet. Risiken der Histamin-Anwendung alleine sind in der Literatur nicht zu finden; allerdings zeigen Daten, dass das Risiko für systemische Nebenwirkungen bei intrakutanen Allergietests bei 0.008% liegt.[14] Da hierbei eine Reihe von Allergenen gleichzeitig mit der Histamingabe untersucht werden, darf man davon ausgehen, dass das isolierte Risiko von Histamin deutlich darunter liegt.

Die afrikanische Juckbohne wird verbreitet zur Herstellung von Juckpulver eingesetzt. Zum Cowhagen-Modell wurden in der bisherigen Literatur keine systemischen Nebenwirkungen berichtet.

Die LLLT wird in der Literatur als nebenwirkungsfrei beschrieben.[2, 15-17]

Die Applikation wird durch Fachärzte für Anästhesiologie und Intensivmedizin in Räumlichkeiten mit Möglichkeiten für Monitoring und Notfallsversorgung durchgeführt.

2. Bezeichnung und Beschreibung des Prüfproduktes

a) *Übersichtsbeschreibung des Prüfprodukts und seiner vorgesehenen Verwendung.*

Der REPULS 7 arbeitet mit kaltem Rotlicht im Wellenlängenbereich von 630nm. Diese Strahlungsform arbeitet auf einer Wellenlänge, welche eine wesentlich höhere Eindringtiefe ermöglicht als UV-Licht. Durch die hohe Leistungsdichte und Nichtlinearität der Dipolkräfte kommt es im Gewebe über Erregung organischer Moleküle, die als Botenstoffe dienen, zu einer Frequenzverdoppelung und somit zu einer Wellenlängenhalbierung. Dies spricht die Resonanz des Botenstoffes an und verändert diesen durch die entstandene UV-Resonanzschwingung am jeweiligen Ort im Gewebe derart, dass Spaltprodukte entstehen und über den Blutkreislauf abtransportiert werden. Somit können entzündliche Veränderungen abklingen.
Der REPULS 7 wird zur Förderung der Wundheilung (Bestrahlung von Wunden nur bei Nicht-Kontaktanwendung und Gebrauch des Distanzrings) und zur Förderung der Narbenheilung verwendet.

b) *Angaben zum Hersteller des Produkts.*

REPULS Lichtmedizintechnik GmbH, Lemböckgasse 61 1230 Wien

c) *Bezeichnung oder Nummer des Modells/Typs Nummer einschließlich der Software-Version und, falls vorhanden der Zubehörteile, um eine vollständige Identifizierung zu ermöglichen.*

Repuls7
Seriennummer: 170670829

d) *Beschreibung, wie die Rückverfolgbarkeit während und nach der Prüfung erreicht werden kann, z. B. Zuweisen von Losnummern, Chargennummern oder Seriennummern.*

Die Klinik für Spezielle Anästhesiologie Schmerz – und Intensivmedizin hat Zugriff zu 2 Geräten, welche auch in der Studie ihren Einsatz finden. Allerdings wird nur ein Gerät (Seriennummer: 170670829) in Betrieb genommen. Das andere Gerät (Seriennummer: 131270190) dient nur zu Scheinbestrahlung.

e) *Vorgesehene Zweckbestimmung des Prüfprodukts in der geplanten klinischen Prüfung.*

In der hier geplanten Prüfung soll der Einfluss der LLLT sowohl auf histaminergen als auch auf nicht-histaminergen Juckreiz bei gesunden Proband*innen geprüft werden.

f) *Populationen und Indikationen, für die das Prüfprodukt vorgesehen ist.*

Der REPULS 7 wird zur Förderung der Wundheilung (Bestrahlung von Wunden nur bei Nicht-Kontaktanwendung und Gebrauch des Distanzrings) und zur Förderung der Narbenheilung verwendet.

g) Beschreibung des Prüfprodukts, einschließlich aller seiner Materialien, die mit den Geweben oder mit Körperflüssigkeiten in Kontakt kommen werden (diese muss Detailangaben über Arzneimittel, menschliches oder tierisches Gewebe oder deren Ableitungen oder sonstige biologisch aktive Substanzen einschließen).

- Bestrahlungseinheit (Artikelnummer 1003): Diese Komponente ist das Kernstück des Gerätes. In der Bestrahlungseinheit befinden sich die Bestrahlungsdioden sowie alle für die Nutzerinteraktion notwendigen Teile: digitales Display, Taster für Eingaben. 2.
- Griff (Artikelnummer 2006): Der Griff ist das Schlüsselelement zur Inbetriebnahme des Gerätes. Ohne diesen Teil kann das Gerät nicht eingeschaltet werden. Der Griff darf nur befugten Personen zugänglich sein!
- Netzteil (Artikelnummer 2020): Die Netzteileinheit versorgt den REPULS 7 mit Strom.
- Schutzbrille (Artikelnummer 2001): Bei der Bestrahlung ist die beiliegende Schutzbrille zu verwenden.
- Distanzring (Artikelnummer 2018): Der Distanzring dient als Blendschutz bei Nichtkontaktanwendungen (Behandlung von Wunden).

h) Übersicht über die erforderliche Schulung und Erfahrung, die für die Anwendung des Prüfprodukts erforderlich ist.

Vor erstmaliger Inbetriebnahme ist eine Schulung für den Anwender erforderlich. Diese umfasst lt. Hersteller folgende Inhalte:

- Technische Daten
- Gerätebedienung &Geräteeinstellung
- Anwendungsrichtlinien & Behandlungsgrundsätze
- Sicherheits- & Gefahrenhinweise
- Fehlerbehandlung
- Wartung & Pflege

Eine diese Inhalte umfassende Schulungsunterlage, die zur persönlichen Einweisung dient, wird jedem Anwender vom Hersteller zur Verfügung gestellt oder kann bei diesem abgerufen werden. Die erfolgreiche Einschulung auf das Gerät wird über ein Einschulungsprotokoll bestätigt. Das Einschulungsprotokoll_Repuls-7 in der aktuellen Version liegt vor und beinhaltet die Bestätigung über die Einschulung der oben genannten Inhalte.

i) Beschreibung der spezifischen ärztlichen oder chirurgischen Maßnahmen, die mit der Anwendung des Prüfprodukts verbunden sind.

Es sind keine spezifischen ärztlichen oder chirurgischen Maßnahmen mit der Anwendung des Prüfprodukts verbunden.

3. Begründung für den Aufbau der klinischen Prüfung

a) *Bewertung der Ergebnisse der relevanten vorklinischen Prüfung/Bewertung, die durchgeführt wurden, um die Verwendung des Prüfproduktes an menschlichen Prüfungsteilnehmern zu begründen, und*

In klinischen Studien konnten positive Effekte von LLLT sowohl auf die Wundheilung, als auch auf die Schmerzverarbeitung nachgewiesen werden,[1-4] welche auf eine Reduktion der neurogenen Inflammation und der damit assoziierten neuronalen Sensibilisierung zurückgeführt werden. Somit kann eine LLLT auch bei akuten und chronischen Schmerzen eingesetzt werden.

b) *Bewertung der klinischen Daten, die für die vorgeschlagene klinische Prüfung relevant sind.*

Im Zuge einer neurogenen Inflammation kommt es auch zu einer Histaminliberation, welche mit Juckreiz vergesellschaftet sein kann Die Autoren dieser hier vorgelegten Studie erwarten somit auch einen positiven Effekt einer LLLT auf Juckreiz.

4. Risiken und Nutzen des Prüfprodukts und der klinischen Prüfung

a) *Voraussichtlicher/vorhersehbarer klinischer Nutzen.*

Die von der vorgelegten Studie erwarteten Erkenntnisse sind von hoher Relevanz für die Beurteilung des medizinischen Nutzens der LLLT in der Juckreiztherapie, da derartige, quantitative Aussagen bisher in der Literatur nicht verfügbar sind. Damit ist zu erwarten, dass diese Studie einen wesentlichen Beitrag für eine fundierte therapeutische Empfehlung bieten kann.

b) *Voraussichtliche/vorhersehbare unerwünschte Wirkung(en) des Produkts.*

Die LLLT wird als nebenwirkungsfrei beschrieben.
Auch sind vom Hersteller keine unerwünschten Wirkungen beschrieben.

c) *Risiken im Zusammenhang mit der Teilnahme an der klinischen Prüfung*

Da nur gesunde Probanden zu Prüfung herangezogen werden und die LLLT als nebenwirkungsfrei beschrieben wird, sind hinsichtlich der LLLT keine Risiken zu erwarten.
Hinsichtlich der Histamin- und Mucuna pruriens Applikation treten vorübergehend Juckreiz und leichte Hautrötungen (zu Studienzwecken beabsichtigt) auf. Diese bleiben lokal auf das Areal der Anwendung der Histamin-Applikation beschränkt und können bis zu 2,5 h anhalten.

d) *Mögliche Wechselwirkungen mit gleichzeitig einhergehenden medizinischen Behandlungen.*

Es sind keine Wechselwirkungen zu erwarten.

e) Zu ergreifende Maßnahmen, um die Risiken zu beherrschen oder abzuschwächen

Keine

f) Risiko-/Nutzen-Begründung.

Ein gesundheitliches Risiko für die teilnehmenden Probanden ist nach heutigem Kenntnisstand faktisch auszuschließen: In der Literatur werden keine unerwünschten Wirkungen beschrieben. Somit ist bei kurzer einmaliger Anwendung innerhalb der Studie ein Risiko durch die LLL-Bestrahlung nach heutigem Kenntnisstand nicht zu erwarten. [18]

5. Ziele und Hypothesen der klinischen Prüfung

a) Primäre und sekundäre Ziele.

Diese Studie soll erstmals unter kontrollierten Bedingungen eine quantitative Analyse des möglichen Einflusses von LLLT sowohl auf histaminergen als auch nicht-histaminergen Juckreiz, Alloknesie und Hyperknesie ermöglichen.

In dieser Studie sollten folgende Fragestellungen beantwortet werden:

- Kann durch die Therapie mit LLL die Intensität des Juckreizes nach Histamin-Applikation vermindert werden?
- Kann durch die Therapie mit LLL die Intensität des nicht-histaminergen Juckreizes nach Mucuna pruriens Applikation vermindert werden?
- Hat die Therapie mit LLL einen Einfluss auf die neurogene Inflammation beim histaminergen Juckreizmodell?
- Hat die Therapie mit LLL einen Einfluss auf die neurogene Inflammation beim nicht-histaminergen Juckreizmodell?
- Hat die Therapie mit LLL einen Einfluss auf die Größe des Alloknesieareals beim histaminergen Juckreiz?
- Hat die Therapie mit LLL einen Einfluss auf die Größe des Alloknesieareals beim nicht-histaminergen Juckreiz?
- Hat die Therapie mit LLL einen Einfluss auf die Größe des Hyperknesieareals beim histaminergen Juckreiz?
- Hat die Therapie mit LLL einen Einfluss auf die Größe des Hyperknesieareals beim nicht-histaminergen Juckreiz?

b) Durch statistische Daten aus der klinischen Prüfung anzunehmende oder abzulehnende Primär- und Sekundärhypothesen.

Primärhypothese:
Durch die Therapie mit LLL verändert sich im Vergleich zu Scheinanwendung die Intensität des Juckreizes nach Histaminapplikation.

Sekundärhypothesen:

- Durch die Therapie mit LLL verändert sich im Vergleich zu Scheinanwendung die Intensität des Juckreizes nach Mucuna pruriens Applikation.
- Durch die Therapie mit LLL verändert sich im Vergleich zu Scheintherapie nach Histamin-Applikation die Größe des Flareareals.
- Durch die Therapie mit LLL verändert sich im Vergleich zu Scheintherapie nach Mucuna pruriens Applikation die Größe des Flareareals.
- Durch die Therapie mit LLL verändert sich im Vergleich zu Scheintherapie nach Histamin-Applikation die Hauttemperatur.
- Durch die Therapie mit LLL verändert sich im Vergleich zu Scheintherapie nach Mucuna pruriens Applikation die Hauttemperatur.
- Durch die Therapie mit LLL verändert sich im Vergleich zu Scheinbehandlung nach Histamin-Applikation die Größe des Alloknesieareals.
- Durch die Therapie mit LLL verändert sich im Vergleich zu Scheinbehandlung nach Mucuna pruriensApplikation die Größe des Alloknesieareals.
- Durch die Therapie mit LLL verändert sich im Vergleich zu Scheinbehandlung nach Histamin-Applikation die Größe des Hyperknesieareals.
- Durch die Therapie mit LLL verändert sich im Vergleich zu Scheinbehandlung nach Mucuna pruriens Applikation die Größe des Hyperknesieareals.

6. Aufbau der klinischen Prüfung

6.1 Allgemeines

a) *Beschreibung des Typs der durchzuführenden Prüfung (z. B. Doppelblind-Vergleichsprüfung, Paralleldesign, mit oder ohne Vergleichsproduktgruppe) mit Begründung für die Auswahl;*

Die Prüfung wird prospektiv, doppelblind, randomisiert und Schein-kontrolliert durchgeführt.

b) *Beschreibung der zu ergreifenden Maßnahmen, um Verzerrungen zu minimieren oder zu vermeiden; einschließlich Randomisierung und Verblindung.*

Die Seite der LLLT bzw. Scheinbehandlung wird durch eine computergenierte Zufallsliste „www.randomizer.at“ bestimmt. Die Behandlung wird durch eine, in der weiteren Studiendurchführung nicht involvierten Person durchgeführt.
Die Proband*innen sind durch eine audiovisuelle Abschirmung (Schwimmbrille mit geschwärzten Gläsern, Gehörschutz) bezüglich der Bestrahlungsseite verblindet. Die

Untersuchenden haben keinen Zugang zur online-Randomisierung und befinden sich während der Bestrahlung nicht im gleichen Raum. Dadurch sind auch sie bezüglich der Bestrahlungsseite verblindet.

c) Primäre und sekundäre Endpunkte mit der Begründung für deren Wahl und Messung.

1. Der histaminerge Juckreiz wird mittels einer Visuellen Analogskala (0-100) ermittelt.
2. Der nicht-histaminerge Juckreiz wird mittels einer Visuellen Analogskala (0-100) ermittelt.
3. Die neurogene Inflammation des histaminergen Juckreizes wird über die Erfassung des Flareareals und über die Messung der Hauttemperatur ermittelt.
4. Die neurogene Inflammation des nicht-histaminergen Juckreizes wird über die Erfassung des Flareareals und über die Messung der Hauttemperatur ermittelt.
5. Ein Alloknesieareal wird beim histaminergen Juckreizmodell mit Hilfe von Wattestäbchen ausgetestet.
6. Ein Alloknesieareal wird beim nicht-histaminergen Juckreizmodell mit Hilfe von Wattestäbchen ausgetestet.
7. Ein Hyperknesieareal wird beim histaminergen Juckreizmodell mit einem von Frey Filament geprüft.
8. Ein Hyperknesieareal wird beim nicht-histaminergen Juckreizmodell mit einem von Frey Filament geprüft.

Juckreizmodelle lassen sich in histamininduzierte und nicht-histamininduzierte unterscheiden. Bisher gab es noch keine strukturierte Untersuchung über die antipruretische Wirkung von LLL, ebenso ist offen, inwieweit der Juckreiz auslösende Mechanismus einen Einfluss auf die suszipierte Wirksamkeit von LLL hat.

Chronischer Juckreiz ist in Analogie zu chronischem Schmerz ebenso häufig mit somatosensorischen Auffälligkeiten vergesellschaftet. Juckreiz-assoziierte Dysästhesien, wie die mechanische Alloknesie und Hyperknesie können als Hinweis für eine neuronale Sensibilisierung bei Prurituspatinent*innen gesehen werden[6] und somit Hinweise liefern ob die antipruretische Wirkung auch bei chonischem Pruritus zu erwarten ist.

d) Verfahren und Wahl des Zeitpunktes für die Bewertung, Aufzeichnung und Analyse der Variablen.

Erhobene Parameter

Intensität des Juckreizes

Die Intensität des Juckreizes wird mittels einer Visuellen Analogskala (0-100), auf welche die Proband*innen zuvor eingeschult wurden, erfasst.

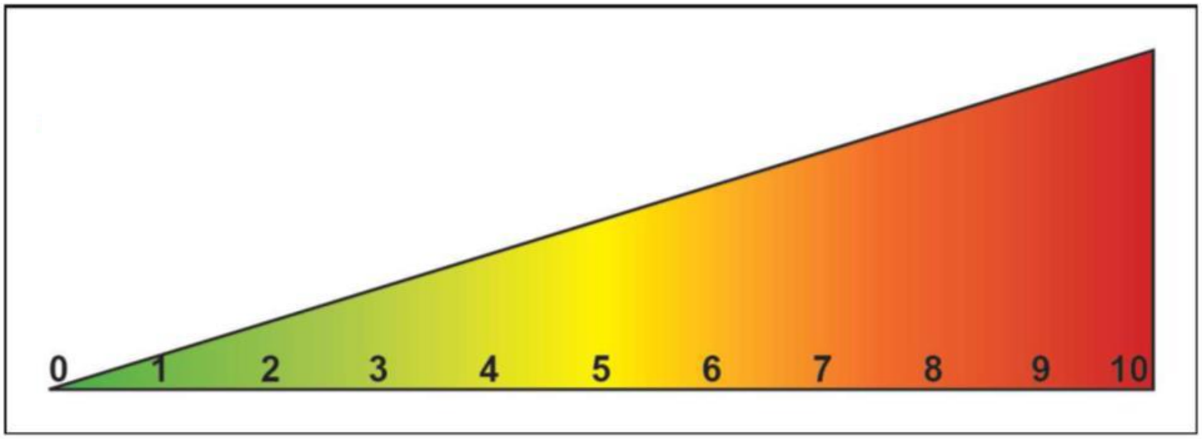

Abbildung 4: VAS Skala des LKH Universitätsklinikum Graz

Flare Areal

Die Kalkulation der Größe des Flare-Areals erfolgt mittels der ImageJ Software (National Institutes of Health, Bethesda, USA; ImageJ is in the public domain: http://rsb.info.nih.gov/ij).

Hauttemperatur

Die Hauttemperatur wird im Zent   rum des Flare Areals mittels einer Corpuls C3 Einmal-Temperatursonde gemessen.

Alloknesie

Es folgt die Erfassung der Alloknesiedistanz mittels eines Wattestabs, mit welchem die Haut entlang von 8 radialen Bahnen in einer Reihe von kurzen Strichen gestreichelt wird. Das Streicheln beginnt 10 cm von der Einstichstelle /Mitte der Cowhageapplikation entfernt und wird in Richtung der Einstichstelle fortgesetzt, bis die Testperson ein Gefühl von Juckreiz während des Streichens meldet oder bis die Striche bis auf 1 cm an die Einstichstelle heranreichen.[12] Die 8 Messungen werden gemittelt.

Hyperknesie

Es folgt die Erfassung der Hypeknesie mit Hilfe eines von Frey Filaments: dies wird ebenso 10 cm von der Einstichstelle/ Mitte der Cowhageapplikation entfernt zentripetal entlang von 8 radialen Linien in 0,5 cm Abständen angesetzt. Die Probanden werden angewiesen, das anfängliche Stechen zu ignorieren und nur das Ausmaß des auftretenden Juckreizes auf einer Visuellen Analog Skala zu beurteilen. Die 8 Messungen werden gemittelt.[12]

Zeitliche Abfolge

Nach der Proband*innenrekrutierung wird eine Aufklärung über die geplante Prüfung durch einen ärztlichen Studienmitarbeiter durchgeführt, gefolgt von der Unterfertigung des Informed Consent Forms durch die Proband*in und den Studienmitarbeiter. Probandinnen werden einem Schwangerschaftstest unterzogen.

Weiters folgt eine computergestützte Randomisierung.

Die Einhaltung der zeitlichen Abfolge wird durch eine Stoppuhr mit vorprogrammierten Signalen koordiniert.

Die Applikation von LLL wird für 12 Minuten mittels Repuls 7 (Repuls Lichtmedizintechnik GmbH, Wien) appliziert.

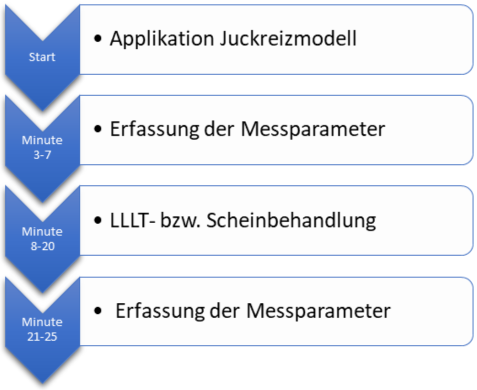

Nach einer Stunde Wiederholung des Algorithmus mit dem jeweils anderem Juckreizmodell

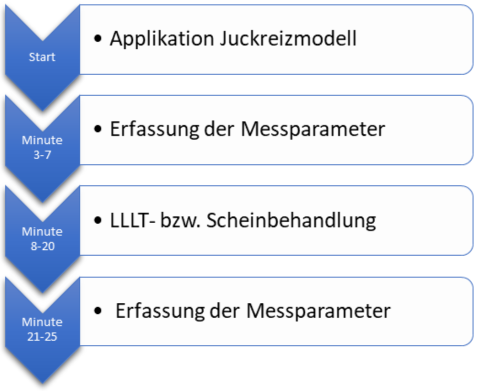

e) Jede Anweisung für den Austausch von Prüfungsteilnehmer

Es kommt zu keinem Austausch der Prüfungsteilnehmer*innen.

6.2 Produkte und Vergleichsprodukte

a) *Beschreibung der Exposition zu den Prüf- oder Vergleichsprodukten, wenn letztere vorgesehen sind.*

Die Proband*innen sind für 2x12 Minuten der Applikation von LLL ausgesetzt (histamininduziertes und nicht-histamininduziertes Pruritusareal). Das Gerät wird mittels eines Abstandsringes 7 cm über dem Pruritusareal angelegt.
Bei der Scheinbehandlung wird das zweite Gerät ebenso mittels Abstandsring über dem Pruritusareal angelegt, jedoch nicht aktiviert.

b) Begründung zur Wahl von Vergleichsprodukten.

keine

c) Aufstellung aller, während der Prüfung anzuwendenden sonstigen Produkte oder medikamentösen Behandlungen.

Die Proband*innnen durchlaufen zwei Juckreizmodelle. Dazu wird der Rücken in 4 Quadranten eingeteilt. Das Histamin Modell wird auf den beiden oberen Quadranten appliziert, das Mucuna pruriens Modell nach einer Stunde Abstand auf den beiden unteren Quadranten.

Histamin Modell
Die Methodik entspricht dem von Darsow et al. beschriebenen Modell.[10] Nach dem Auftragen eines Tropfens Histamingel (1% Histamin-Dihydrochlorid in 2,5% Methylcellulose) wird die Haut mit Hilfe von herkömmlichen Blutlanzetten, wie sie in der Allergiediagnostik üblich sind, oberflächlich punktiert.[10] Die Applikation wird immer von demselben Studienmitarbeiter durchgeführt, um die Variabilität in der Applikationstechnik zu minimieren.

Cowhagen/Mucuna pruriens Modell
Die Pflanzenhaare der Mucuna pruriens (afrik. Juckbohne) sind ein etabliertes Modell, um einen histamin-unabhängigen Juckreiz zu induzieren. Die Methodik entspricht dem von Papoiu et al. beschriebenen Modell. Mittels Hansaplaststreifen wird ein Areal von 2x2cm umklebt, um ein Ausbreiten der Pflanzenhaare auf die umliegende Haut zu vermeiden. In diesem Hautareal werden 40 bis 45 Fäden, welche zuvor unter dem Mikroskop gezählt werden, mit einer Mikropinzette aufgebracht und 45 Sekunden lang sanft eingerieben.[11]

6.3 Prüfungsteilnehmer

a) Einschlusskriterien für die Auswahl der Prüfungsteilnehmer

Gesunde Proband*innen im Alter von 18-60 Jahren, die nach ausführlicher Aufklärung zur Mitwirkung bereit sind (unterschriebener Informed Consent). Bei Probandinnen negativer Schwangerschaftstest.
.

b) Ausschlusskriterien für die Auswahl der Prüfungsteilnehmer.

- Bekannte Allergie oder Überempfindlichkeit gegen Histamin oder die afrikanische Juckbohne
- anamnestische Vorliegen von Hautkrankheiten
- Piercings
- Fieber
- Tattoos im Prüfgebiet
- Neoplasie im Prüfgebiet
- Schwangerschaft
- Schrittmacher
- Lokal akuter Infekt, Hautentzündungen,- ausschläge

- Epilepsie

c) *Aufnahmezeitpunkt.*

Unterfertigung des Informed consents

d) *Erwartete Gesamtdauer der klinischen Prüfung.*

3 Wochen

e) *Erwartete Teilnahmedauer jedes Prüfungsteilnehmers.*

3 Stunden

f) *Geforderte Anzahl der in die klinische Prüfung aufzunehmenden Prüfungsteilnehmer.*

17 Proband*Innen

g) *Geschätzter Zeitaufwand zur Gewinnung dieser Personenzahl (d. h. Aufnahmedauer).*

Die Rekrutierungsphase wird sich in etwa über 2 Wochen erstrecken. Die Rekrutierung erfolgt mittels Aushangs an Aushangbretter der ÖH an der KF-Uni Graz (Hauptgebäude, Resowi, Wall-Gebäude).

6.4 Behandlungen

a) *Beschreibung aller Behandlungen, die während der klinischen Prüfung an den Prüfungsteilnehmern durchgeführt werden.*

Die Applikation von LLL wird für 12 Minuten mittels Repuls 7 (Repuls Lichtmedizintechnik GmbH, Wien) appliziert. Das Gerät wird mittels eines Distanzrings 7cm von der Haut entfernt positioniert.
Die Scheinbehandlung wird mit dem gleichen Gerät und dem gleichen Distanzring durchgeführt, ohne dass dieses aktiviert wird.

b) *Alle bekannten oder vorhersehbaren Faktoren, die die Ergebnisse der klinischen Prüfung oder deren Interpretation beeinträchtigen können.*

Die Ergebnisse, die in dieser Prüfung in einem Kollektiv gesunder Probanden und einem experimentellen Juckreizmodell unter Einmalanwendung der LLLT erzielt werden, können nur eingeschränkt auf die klinische Anwendung übertragen werden.

6.5 Festlegungen für das Monitoring

Die Prüferin/der Prüfer erklärt sich damit einverstanden, dass die für das Monitoring verantwortliche Person gemäß Monitoringplan eine Datenüberprüfung vornimmt, um die zufriedenstellende Erhebung der Daten und die Einhaltung des Prüfplanes zu sichern.
Weiterhin erklärt sie bzw. er sich zur Zusammenarbeit mit dieser Person bereit und dazu, ihr, wann immer nötig, alle erforderlichen Informationen zukommen zu lassen. Dies beinhaltet den Zugang zu allen Unterlagen, die mit der Prüfung im Zusammenhang stehen, einschließlich der prüfungsrelevanten Patientenakten im Original. Zu den Aufgaben der Prüferin/des Prüfers zählt dabei, die Patientenakte so vollständig wie möglich zu führen, d. h., Informationen zu Krankengeschichte, Begleiterkrankungen, Aufnahme in die Prüfung, Besuchsdaten, Ergebnisse von Untersuchungen, Medikamentenausgabe sowie Unerwünschte Ereignisse festzuhalten. Der Monitorin/dem Monitor wird außerdem ermöglicht, die Datenüberprüfung sowie den Vergleich mit den relevanten Patientenakten gemäß den SOPs und den ICH-GCP-Richtlinien in den vorher festgelegten Zeitabständen durchzuführen, um die Einhaltung des Prüfplans und die kontinuierliche Aufzeichnung der Daten zu gewährleisten. Dabei werden alle medizinischen Originalbefunde, die als Quelle für die Informationen im CRF bzw. in der Datenbank notwendig sind, überprüft. Die Prüfungsteilnehmer haben sich durch Unterzeichnung der Einwilligungserklärung mit einer solchen Überprüfung einverstanden erklärt.
Die für das Monitoring verantwortliche Person ist verpflichtet, alle Informationen vertraulich zu behandeln und den grundsätzlichen Anspruch der teilnehmenden Personen auf Integrität und Schutz ihrer Privatsphäre zu wahren.

7. Statistische Überlegungen

a) statistische Planung, das Verfahren und die analytischen Verfahren,

Die Fallzahlberechnung wurde basierend auf folgenden Annahmen durchgeführt: In der Publikation von Arendt-Nielsen et al. wurde Juckreizstärke mit 32.9 ± 5.6 ermittelt. Eine Reduktion von mehr als 15% wurde als klinisch relevant definiert.

Statistische Auswertung
Die statistische Auswertung erfolgt - abhängig von der Verteilung - mittels 2-seitigem T-Test für abhängige Stichproben oder Wilcoxon Test. Das Signifikanzniveau alpha wird mit 0.05 definiert.

b) die Stichprobengröße,

Daraus ergibt sich für einen 2-seitigen T-test mit verbundenen Stichproben eine minimale Stichprobengröße von 15 Probanden. Die Berechnung der Stichprobengröße wurde mit G*Power 3.1.9.5 durchgeführt.[13]

c) das Signifikanzniveau und die Aussagekraft der klinischen Prüfung,

Das Signifkanzniveau alpha wurde mit 0.05 definiert, beta mit 0.1.

d) die zu erwartenden Ausfallraten,

Um Drop-outs berücksichtigen zu können, wird die Stichprobengröße auf 17 Probanden erhöht.

e) die auf die Ergebnisse der Prüfung anzuwendenden Bestehens-/Versagenskriterien,
keine

f) gegebenenfalls die Maßnahmen für eine Zwischenauswertung,
keine

g) die Kriterien für einen aus statistischen Gründen notwendigen Abbruch der Prüfung,
keine

h) Anweisungen für den Bericht über alle Abweichungen von der ursprünglichen statistischen Planung,
keine

i) die Spezifikation der Untergruppen der Analyse,
keine

j) Verfahren, die alle Daten in Betracht ziehen,
keine

k) die Behandlung fehlender, nicht verwendeter oder falscher bzw. irreführender Daten, einschließlich von Ausfällen und Zurückziehungen einzelner Prüfungsteilnehmer,
keine

l) eine Begründung für die Nichtberücksichtigung einzelner Informationen bei der Hypothesenprüfung, sofern zutreffend, und
keine

m) bei multizentrischen Prüfungen, die Mindest- und Höchstzahl an einzubeziehenden Prüfungsteilnehmern für jedes Prüfzentrum.
keine

8. Datenmanagement

Jedem Probanden wird ein Studiencode zugeteilt. Die Studienunterlagen werden versperrt aufbewahrt. Nur autorisiertes Personal hat Zugriff auf diese Daten.

Die Datenanalyse erfolgt nur anhand der Studiencodes.

Die Bestimmungen der Datenschutzgrundverordnung werden beachtet.

Entsprechend dem KaKuG werden die Krankengeschichten 30 Jahre aufbewahrt.

9. Änderungen am klinischen Prüfplan

Das Votum der Ethikkommission deckt nur die im Antrag enthaltenen Angaben ab, umfasst also nicht die zu einem späteren Zeitpunkt vorgenommenen Ausweitungen und Änderungen des Forschungsvorhabens. Bei Änderungen ist ein Prüfplananhang (Amendment) nötig, welcher vom Prüfer unterschrieben werden muss. Jede Änderung des Prüfplans muss zu jedem sich in Umlauf befindlichen Prüfplan als Amendment angefügt werden. Alle substantiellen Prüfplanänderungen sind der Ethikkommission mitzuteilen. Bei Prüfplanänderungen, die nicht ausschließlich formellere Natur sind und für den *Prüfungsteilnehmer* relevante Änderungen enthalten, ist ein erneutes Votum der Ethikkommission einzuholen. Auf Änderungen der Studienbedingungen sind die Patienten/Probanden ggf. im Rahmen der Aufklärung und Einwilligung hinzuweisen.
Der Behörde (BASG) müssen bedeutsame Änderungen des Prüfplans ebenfalls gemeldet werden. Für diese Meldungen sind die entsprechenden Meldeformulare auf der BASG Website zu verwenden.

10. Abweichungen vom klinischen Prüfplan

a) Erklärung, die festlegt, dass es dem Prüfer nicht gestattet ist, vom CIP abzuweichen.

Abweichungen vom Prüfplan sind nicht erlaubt, außer im Falle von dringend gebotenen Sicherheitsmaßnahmen für die Prüfungsteilnehmer. Solche Urgent Safety Measures sind unverzüglich nach deren Umsetzung an die Ethikkommission/das BASG zu melden.

b) Anweisungen zum Aufzeichnen, Melden und Analysieren von Abweichungen vom klinischen Prüfplan.

Prüfplanabweichungen werden vom PI dokumentiert und eine Ursachenanalyse durchgeführt, anhand derer Maßnahmen zur zukünftigen Vermeidung der Abweichung (Korrektur- und Vorbeugemaßnahmen) implementiert werden.

Prüfplanabweichungen, welche einen Einfluss auf die Patientensicherheit haben könnten, werden an die Ethikkommission gemeldet.

c) Anforderungen an die Meldung und den zeitlichen Rahmen.

Die Meldung sollte, wenn zutreffend, zeitnahe nach Bekanntwerden der Abweichung erfolgen.

11.

12. Rechtliche Grundlagen

12.1
Bei der Durchführung der Studie sind neben der Deklaration von Helsinki (in der jeweils geltenden Fassung) folgende Richtlinien und Gesetze zu beachten: z.B.

• MPG in der jeweils geltenden Fassung
• EN 14155
• ICH-GCP Guideline
• EU- REGULATION (EU) 2017/745

12.2
Gemäß Medizinproduktegesetz wird eine Personenschaden-/Rechtsschutz-Versicherung abgeschlossen.

12.3 Votum der Ethik-Kommission

Die klinische Prüfung darf nur begonnen werden, wenn die zustimmende Stellungnahme der zuständigen Ethikkommission und die Nichtuntersagung / Genehmigung der zuständigen Behörde (BASG) vorliegen.

13. Verfahren zum Einholen der Einverständniserklärung

a) *Beschreibung des allgemeinen Verfahrens zum Einholen der Einverständniserklärung einschließlich des Verfahrens die Prüfungsteilnehmer mit neuen Informationen zu versorgen, wenn notwendig.*

Probanden werden durch autorisierte Studienmitarbeiter aufgeklärt. Die Probanden haben ausreichend Zeit, ihre Entscheidung zu treffen und Fragen zu stellen. Die Probanden erhalten eine Kopie der Aufklärung. Sollten relevante neue Informationen zur Verfügung stehen, erfolgt eine neuerliche Aufklärung der Probanden, welche noch in der Studie sind.

14. Unerwünschte Ereignisse, unerwünschte Wirkungen des Produkts und Produktmängel

a) Definitionen unerwünschter Ereignisse

Ein **unerwünschtes Ereignis (Adverse Event, AE)** bezeichnet ein nachteiliges medizinisches Ereignis, eine nicht vorgesehene Erkrankung oder Verletzung oder nachteilige klinische Symptome, einschließlich anormaler Laborbefunde, bei Prüfungsteilnehmern, Anwendern oder anderen.

Ein **AE** kann daher jede ungünstige und unbeabsichtigte Reaktion (einschließlich eines anomalen Laborbefunds), jedes Symptom oder jede vorübergehende Erkrankung sein, ob dieses nun mit dem Prüfprodukt in Verbindung steht oder nicht.

Eine **unerwünschte Wirkung des Produkts** bezeichnet ein AE, bei dem ein kausaler Zusammenhang zum Studienprodukt/dem Prüfverfahren angenommen wird.

b) Definitionen schwerwiegender unerwünschter Ereignisse

Ein schwerwiegendes unerwünschtes Ereignis (**Serious Adverse Event, SAE**) bezeichnet ein unerwünschtes Ereignis, das eine der nachstehenden Folgen hatte:

a) Tod

b) schwerwiegende Verschlechterung des Gesundheitszustands des Prüfungsteilnehmers, die ihrerseits eine der nachstehenden Folgen hatte: i) lebensbedrohliche Erkrankung oder Verletzung,

ii) bleibender Körperschaden oder dauerhafte Beeinträchtigung einer Körperfunktion, iii) stationäre Behandlung oder Verlängerung der stationären Behandlung des Patienten, iv) medizinische oder chirurgische Intervention zur Verhinderung einer lebensbedrohlichen Erkrankung oder Verletzung oder eines bleibenden Körperschadens oder einer dauerhaften Beeinträchtigung einer Körperfunktion, v) chronische Erkrankung,

c) Fötale Gefährdung, Tod des Fötus oder kongenitale körperliche oder geistige Beeinträchtigungen oder Geburtsfehler

Eine **schwerwiegende unerwünschte Wirkung des Produkts (SADE)** bezeichnet ein SAE, bei dem ein kausaler Zusammenhang zum Studienprodukt/dem Prüfverfahren angenommen wird.

c) Definition Produktmängel

„Produktmangel“ bezeichnet eine Unzulänglichkeit bezüglich Identifizierung, Qualität, Haltbarkeit, Zuverlässigkeit, Sicherheit oder Leistung eines Prüfprodukts, einschließlich Fehlfunktionen, Anwendungsfehlern oder Unzulänglichkeit der vom Hersteller bereitgestellten Information

e.) Dokumentations- und Meldepflichten gemäß Medical Device Regulation, Artikel 80

Der Sponsor führt **vollständige Aufzeichnungen** über alle folgenden Elemente:

a) unerwünschte Ereignisse aller Arten, die im klinischen Prüfplan als entscheidend für die Bewertung der Ergebnisse dieser klinischen Prüfung bezeichnet wurden;
Entscheidend für die Bewertung der Ergebnisse wäre das Auftreten von Schmerzen im Prüfgebiet durch die Anwendung der LLLT.
b) alle schwerwiegenden unerwünschten Ereignisse;
c) jeden Produktmangel, der bei Ausbleiben angemessener Maßnahmen oder eines Eingriffs oder unter weniger günstigen Umständen zu schwerwiegenden unerwünschten Ereignissen hätte führen können;
d) alle neuen Erkenntnisse in Bezug auf ein Ereignis gemäß den Buchstaben a bis c.

Der Sponsor **meldet** unverzüglich allen Mitgliedstaaten, in denen die klinische Prüfung durchgeführt wird,

a) jedes schwerwiegende unerwünschte Ereignis, das einen Kausalzusammenhang mit dem Prüfprodukt, dem Komparator oder dem Prüfverfahren aufweist oder bei dem ein Kausalzusammenhang durchaus möglich erscheint,
b) jeden Produktmangel, der bei Ausbleiben angemessener Maßnahmen oder eines Eingriffs oder unter weniger günstigen Umständen zu schwerwiegenden unerwünschten Ereignissen hätte führen können,
c) alle neuen Erkenntnisse in Bezug auf ein Ereignis gemäß den Buchstaben a und b.

Die Frist, innerhalb deren die Meldung zu erfolgen hat, hängt von der Schwere des Ereignisses ab. Ist dies notwendig, um eine zügige Meldung zu sicherzustellen, kann der Sponsor zunächst eine unvollständige Meldung übermitteln und dieser dann die vollständige Meldung folgen lassen. Die Meldung erfolgt ebenso an die beurteilende Ethikkommission.

15. Vorzeitige Beendigung oder Aussetzen der Prüfung

Anforderungen an die Nachbeobachtung der Prüfungsteilnehmer.

Eine Nachbeobachtung der Proband*Innen ist nicht geplant.

15.1 Abbruch der Studie bei einem Probanden (Drop-out)

Einer oder mehrere der folgenden Umstände können z.B. zu einem Abbruch der Studie bei einem einzelnen Probanden führen (dieser Proband wird als drop-out gewertet):
• Rücknahme der Einwilligung des Probanden
• Nichttolerierbare unerwünschte Wirkungen
• Verletzung des Studienprotokolls
• Auftreten eines Ausschlusskriteriums
• Auftreten einer Erkrankung
• Schwangerschaft
• andere Umstände, die die Gesundheit des Probanden gefährden würden, wenn er weiterhin an der Studie teilnimmt.

15.2 Abbruch der gesamten Studie

Der Prüfarzt kann zum Wohl und im Interesse der Probanden die Studie zu jedem Zeitpunkt abbrechen, wenn schwere Nebenwirkungen oder andere unvorhersehbare Umstände auftreten.

16. Veröffentlichungspolitik und Abschlussbericht

Die Studie wird vor ihrem Start auf Clinicaltrials.gov registriert

Alle randomisierten und kontrollierten klinischen Prüfungen, die nach dem 1. Juli 2005 Patientinnen/Patienten rekrutieren, müssen in einer öffentlich zugänglichen Datenbank (z. B. www.clinicaltrials.gov) registriert werden, um nach Vorliegen der Prüfungsergebnisse diese in renommierten Fachzeitschriften veröffentlichen zu können.

Der Abschlussbericht ist ein Jahr nach Beendigung der Studie an das BASG/die Ethikkommission zu übermitteln.

17. Literaturhinweise

Referenzen

1. Esper, M.A., R.A. Nicolau, and E.A. Arisawa, The effect of two phototherapy protocols on pain control in orthodontic procedure--a preliminary clinical study. Lasers Med Sci, 2011. 26(5): p. 657-63.
2. Lang-Illievich, K., et al., The Effect of Low-Level Light Therapy on Capsaicin-Induced Peripheral and Central Sensitization in Healthy Volunteers: A Double-Blinded, Randomized, Sham-Controlled Trial. Pain Ther, 2020.
3. Langella, L.G., et al., Photobiomodulation therapy (PBMT) on acute pain and inflammation in patients who underwent total hip arthroplasty—a randomized, triple-blind, placebo-controlled clinical trial. Lasers in medical science, 2018. 33(9): p. 1933-1940.
4. Lima, A.C.G., et al., Low-level laser and light-emitting diode therapy for pain control in hyperglycemic and normoglycemic patients who underwent coronary bypass surgery with internal mammary artery grafts: a randomized, double-blind study with follow-up. Photomedicine and laser surgery, 2016. 34(6): p. 244-251.
5. Rosa, A.C. and R. Fantozzi, The role of histamine in neurogenic inflammation. Br J Pharmacol, 2013. 170(1): p. 38-45.
6. Andersen, H.H., et al., Alloknesis and hyperknesis-mechanisms, assessment methodology, and clinical implications of itch sensitization. Pain, 2018. 159(7): p. 1185-1197.
7. de Oliveira, R.A., et al., Low-intensity LED therapy (658 nm) on burn healing: a series of cases. Lasers Med Sci, 2018. 33(4): p. 729-735.
8. Bizjak Ogrinc, U., et al., Efficacy of Non-ablative Laser Therapy for Lichen Sclerosus: A Randomized Controlled Trial. J Obstet Gynaecol Can, 2019. 41(12): p. 1717-1725.
9. Andersen, H.H., et al., Nonhistaminergic and mechanical itch sensitization in atopic dermatitis. Pain, 2017. 158(9): p. 1780-1791.
10. Darsow, U., et al., Correlations between histamine-induced wheal, flare and itch. Arch Dermatol Res, 1996. 288(8): p. 436-41.
11. Papoiu, A.D., et al., Cowhage-induced itch as an experimental model for pruritus. A comparative study with histamine-induced itch. PLoS One, 2011. 6(3): p. e17786.
12. Sikand, P., et al., Similar itch and nociceptive sensations evoked by punctate cutaneous application of capsaicin, histamine and cowhage. Pain, 2009. 144(1-2): p. 66-75.
13. Faul, F., et al., Statistical power analyses using G* Power 3.1: Tests for correlation and regression analyses. Behavior research methods, 2009. 41(4): p. 1149-1160.
14. Codreanu, F., et al., The risk of systemic reactions to skin prick-tests using food allergens: CICBAA data and literature review. Eur Ann Allergy Clin Immunol, 2006. 38(2): p. 52-4.
15. Hertz, H., Der Repuls©-Tiefenstrahler als zusätzliche Therapieoption bei Patienten mit Schulterbeschwerden. 2012.
16. de Freitas, L.F. and M.R. Hamblin, Proposed mechanisms of photobiomodulation or low-level light therapy. IEEE Journal of selected topics in quantum electronics, 2016. 22(3): p. 348-364.
17. Hoseinpour Jajarm, H., et al., The effects of photodynamic and low-level laser therapy for treatment of oral lichen planus-A systematic review and meta-analysis. Photodiagnosis Photodyn Ther, 2018. 23: p. 254-260.
18. Rayegani, S.M., et al., Safety and Effectiveness of Low-Level Laser Therapy in Patients With Knee Osteoarthritis: A Systematic Review and Meta-analysis. J Lasers Med Sci, 2017. 8(Suppl 1): p. S12-s19.

18. Unterschriften

18.1

Mit der Unterschrift wird bestätigt, dass die Studie gemäß ICH-GCP, der Deklaration von Helsinki, der nationalen Gesetzgebung und dem vorliegenden Studienprotokoll durchgeführt wird.

Sponsor bzw. dessen Vertreter/in

Dr. Kordula Lang-Illievich **Graz, 3.6.2**

18.2

Prüferin/Prüfer

Hiermit bestätige ich, dass ich den vorliegenden Prüfplan gelesen und verstanden habe und in allen Teilen anerkenne. Ich verpflichte mich, dafür zu sorgen, dass die von meinem Zentrum in die Prüfung eingebrachten Personen nach den Festlegungen dieses Prüfplans behandelt, beobachtet und dokumentiert werden.

Dr. Kordula Lang-Illievich **Graz, 3.6.21**

.
