# Supplementary material for: The effect of photobiomodulation on histamine and Mucuna pruriens-induced pruritus, hyperknesis and alloknesis in healthy volunteers: A double-blind, randomized, sham-controlled study
Source: PLoS One. 2024 Jul 18;19(7):e0307034. doi: 10.1371/journal.pone.0307034 (PMC11257285; doi:10.1371/journal.pone.0307034)
Supplement: Attachment — Submitted filename: Reviewer Comments.docx [file pone.0307034.s004.docx]

Review Comments to the Author

Reviewer #1:

The analysis, as reported, requires revision particularly the calculation of confidence intervals for the outcome measurers are required.

We have now included the CO intervals.

Lines
30-33 It would have been useful to include the duration of the LLLT/Sham and also the fact that the outcome measures taken were assessed pre- and post- treatment.

We have added these information.

35-42 This will need a total rewrite taking account of the points indicated below
We have rewritten this section.

210 Need to include the units for the scale used for the measure of 32.9
We added this information.

216-220 In a Pre and Post treatment study with paired observations made on each subject, it is not important to check the Pre and Post data individually for the Normal distributions of each. For each patient, it is the change d = Post − Pre which is of interest. Whatever the distribution form of Pre and Post, d will tend to have a Normal distribution. [This is also true if the data are non-parametric.] Thus, the tests discussed here are not relevant.

We have erased this passage and re-calculated the data accordingly.
What is now important is to determine the mean of these differences, ¯d and then do the paired t-test of the null hypothesis of 0 difference to get the p-value.
More essential however, is to calculate and report the corresponding 95% Confidence Interval (CI).
We are now reporting the results according to the paired-t-test including the 95%-CI of the difference.

226 According to Table 1 this should be Type VI

We have corrected this issue.

230 Table 1: For describing characteristics of the patients, it is better to quote (say) the mean, age with youngest and oldest patient ages rather than SD. Similarly for height, and weight
We have now included minimum and maximum values for these measures.

230 Table 1: Would it be better to replace ‘Race, White’ by ‘Fair skin Caucasian’?

We have changed the wording

230 Table 1: Suggest omit: Types I, II, III, IV and VI
We have omitted the unnecessary types.

233-247 This will need a total rewrite taking account of some of the points raised

We have rewritten this paragraph.

251-252 Table 2: The difference d = LLLT – Sham, needs presenting with its 95% CI, and the p-value. For example, Histamine VAS: d = 30.5 − 16.6 =13.9 with 95%CI XX to YY, p = 0.00001.[I cannot calculate the CI or p-value without the 13 paired differences.]
We have now reported the results according to these suggestions.

251-252 Table 2: ‘Itch’ should be added to the second row
We added the word itch.

251-252 Table 2 and elsewhere: P-values should be quoted to two significant places. So, for example, ‘0.75774 and 0.07960’ become ‘0.76 and 0.080’ respectively

We have changed the reporting or the p-values throughout the manuscript.

# Reviewer #2:

Materials and methods sections
1.As for LLLT intervention, is there any shelter or other methods to eliminate sparing effect between quadrants?

The spacer ring was opaque and prevented accidental irradiation of surrounding tissue. We added this information to the methodology.

2.Previous studies has demonstrated the acute itch evoked by whether histamine or cowhage only lasts for 5-20 minutes. How long the induced-itch last in this trial? And please clarify whether the itch sensation subside itself or reduced by LLLT? [Andersen HH et al. Alloknesis and hyperknesis-mechanisms, assessment methodology, and clinical implications of itch sensitization. Pain. 2018;159(7):1185-1197.]
As we have not assessed these data overtime, we cannot provide it. We have however added this point in the limitation section.

3.The calculation of simple size has been presented in supporting information. I recommend that it should be included in the manuscript.

The sample size calculation is now in the statistics section.

Discussion section
1.The 3rd paragraph presented interpretation of the effect of LLLT in reducing itch, however, only its influence on mast cell was discussed. I suggest authors should provide with more evidence on more key factors in pruritus pathway, including inflammatory and nonhistamine factors.

We now have broadened this section and included considerations on non-histamine factors.

2. There is only 1 session of LLLT administered to subjects. I recommend that the extent of clinical significance of these result should be discussed.

We added this point in the “future perspective section”.

Typos
1.Line 27. It should be “hyperknesis”, rather than “hyperkinesis”. There are some of the same typo in this manuscript which should be revised.

We have corrected this issue throughout the manuscript.

2.Line 70. It seems that the reference number “(11)” is large in font size. And some punctuation surrounding the reference number in the main text were not correct.

We have corrected this issue.

3.Table 2. There should be a space between “M. pruritus” and “Alloknesia”.

We have corrected this issue.

4.Line 308. The full comma was missing at the end of this paragraph.

We have corrected this issue.

# Reviewer #3:

Firstly, the terminology used for Low-Level Light Therapy (LLLT) has been updated to photobiomodulation.

We have now added the exprecession in the title, abstract and introduction.

Secondly, the LED device you employed (Repuls 7) exhibits an irradiance level of 175mW/cm², potentially inducing thermal effects in tissues. This level of irradiance exceeds the typical threshold for photobiomodulation, defined as not surpassing 100mW/cm² in the skin. Furthermore, you did not provide measurements of actual irradiance using a power meter from a distance of 7cm from the skin surface. The relationship between irradiance and distance is crucial, following an inverse square law, meaning irradiance decreases as distance from the light source increases. Assuming a power of 4100W at 1cm, the actual irradiance would approximate 83mW/cm², resulting in a fluence of 30J/cm² over 6 minutes—a detail not mentioned in your study. Additionally, your choice to use a pulsing frequency of 2.5Hz raises questions, as a continuous wave (CW) might have been more appropriate to avoid introducing an unverified variable. Beam uniformity, including potential hot and cold spots, was also not addressed.

We concur with the reviewer's assertion that it would have been intriguing to ascertain the precise power output. However, we are unable to provide the requisite data. Nevertheless, we have incorporated this aspect into the limitations section.

Lastly, considering that photobiomodulation effects can be both local and systemic, a washout period of only one hour between procedures seems inadequate. The effects of photobiomodulation are typically observed between 24 to 48 hours post-exposure, suggesting a need for a longer interval.

By using a randomization schema for both, the pruritus model and the treatment order and by switching between four different treatment sides we hoped to minimize this issue. Nevertheless, we agree with the reviewer that there is a risk of a systemic carry-over effect. We have included this in our limitations accordingly.
